# Infrastructure Related Road Safety Interventions

**Published:** 2019-08

**Authors:** Rahim Farahnak Benekohal

**Affiliations:** ^*a*^Professor of Civil and Environmental Engineering, University of Illinois at Urbana-Champaign, Urbana, USA.

## Abstract

Approximately 1.35 million people die on the world's roads every year (That is about 3700 every day!!). Nearly half (49%) of the fatalities are pedestrians, cyclists and motorcyclists. In addition, there are 20-50 million people injured every year due to road traffic. Studies have shown that road design deficiencies largely contribute to road traffic injuries. A holistic and integrated approach to road safety must be taken to improve traffic safety.

From the five Es of traffic safety (engineering, education, enforcement, emergency services, and evaluation) the emphasis is placed on infrastructure design and improvements and their roles on improving traffic safety. Traffic safety is a key factor in planning, designing and operating of a safe road network. Roadway design and traffic operation/management are key components of the 5 Es of improving traffic safety. Roadway design elements and traffic operation and management decision that directly affects roadway safety are discussed. Actions to be taken are presented and effectiveness of infrastructure related road safety intervention are discussed.

**Keywords:**

Road Safety, Infrastructure, Safe Road Network

